# *Foxp2* Mutations Impair Auditory-Motor Association Learning

**DOI:** 10.1371/journal.pone.0033130

**Published:** 2012-03-07

**Authors:** Simone Kurt, Simon E. Fisher, Günter Ehret

**Affiliations:** 1 Institute of Neurobiology, University of Ulm, Ulm, Germany; 2 Max Planck Institute for Psycholinguistics, Nijmegen, Netherlands; 3 Donders Institute for Brain, Cognition and Behaviour, Radboud University, Nijmegen, Netherlands; 4 Wellcome Trust Centre for Human Genetics, University of Oxford, Oxford, United Kingdom; Charité Universitätsmedizin Berlin, NeuroCure Clinical Research Center, Germany

## Abstract

Heterozygous mutations of the human *FOXP2* transcription factor gene cause the best-described examples of monogenic speech and language disorders. Acquisition of proficient spoken language involves auditory-guided vocal learning, a specialized form of sensory-motor association learning. The impact of etiological *Foxp2* mutations on learning of auditory-motor associations in mammals has not been determined yet. Here, we directly assess this type of learning using a newly developed conditioned avoidance paradigm in a shuttle-box for mice. We show striking deficits in mice heterozygous for either of two different *Foxp2* mutations previously implicated in human speech disorders. Both mutations cause delays in acquiring new motor skills. The magnitude of impairments in association learning, however, depends on the nature of the mutation. Mice with a missense mutation in the DNA-binding domain are able to learn, but at a much slower rate than wild type animals, while mice carrying an early nonsense mutation learn very little. These results are consistent with expression of *Foxp2* in distributed circuits of the cortex, striatum and cerebellum that are known to play key roles in acquisition of motor skills and sensory-motor association learning, and suggest differing in vivo effects for distinct variants of the Foxp2 protein. Given the importance of such networks for the acquisition of human spoken language, and the fact that similar mutations in human FOXP2 cause problems with speech development, this work opens up a new perspective on the use of mouse models for understanding pathways underlying speech and language disorders.

## Introduction

The gene *Foxp2* of the forkhead gene family is expressed during the ontogeny of the mammalian brain in areas such as the deep layers of the cortex, medium spiny neurons of the basal ganglia, parts of the thalamus, and the Purkinje cells of the cerebellum [1]–[9]. Foxp2-expressing neurons in these structures belong to distributed circuits involved in motor coordination, procedural learning and acquisition of motor skills, and sensory-motor integration and learning [3], [6], [8], [10]–[13]. Such brain circuits are also of crucial importance for learning the complex orofacial and laryngeal movements for speech production and for reaching language competence [14]–[17]. Indeed, heterozygous mutations of the *FOXP2* gene in humans cause severe speech and language disorders [1], [3], [18]–[20], functional knockdown of *FoxP2* in young zebra finches causes incomplete and inaccurate vocal imitation during song learning [11], [20], and heterozygous etiological mutations of the *Foxp2* gene in mice impair the acquisition of motor skills [12], [20] without overt effects on innately produced vocalizations of neonate mouse pups [21]. Together, these data led us to hypothesize that the effects of *Foxp2* mutations on motor coordination might become most apparent in the context of auditory-motor learning, i.e. learning different motor patterns in association with the perception of different sounds.

The present study was designed to test this hypothesis. There is little evidence that mice learn their vocalization patterns, although the properties of their calls depend on variables such as genetic background, age, gender, motivation, and environmental factors [22]–[27]. Thus, we used an alternative paradigm for studying auditory-motor learning in mice, one that allows the discrimination between improvement of motor performance (acquisition of a motor skill) and improvement of auditory-motor associations to establish a cognitive skill. In particular, we applied a recently developed shuttle-box paradigm for mice that measures learning of both motor skills and auditory-motor associations. In this paradigm, motor-skill learning is quantified by observations of the animal crossing a hurdle, which separates two compartments of a box. Auditory-motor association learning is measured by quantifying the speed and the performance level of associating a certain tone with the requirement of hurdle crossing and another tone with staying where you are [28]. We studied these aspects of learning in mice carrying heterozygous *Foxp2* mutations that are similar to those implicated in human speech and language disorders. We show that besides motor-skill learning, auditory-motor association learning is impaired by these heterozygous *Foxp2* mutations, and we demonstrate that the strength of the effect depends on the type of the mutation.

## Results and Discussion

Auditory-motor association learning was assessed by training mice to associate distinct response behaviors with perception of different tone frequencies in a shuttle-box [28]: animals learned to jump across the hurdle separating the two shuttle-box compartments in response to 12 kHz tones and to remain in the compartment where they were when hearing 7 kHz tones. The jump across the hurdle is a motor skill, the jump to the correct tone is a cognitive skill acquired through auditory-motor association learning.

We focused on two distinct mutations affecting mouse *Foxp2*, each of which has clear relevance for human speech disorders [12]. The R552H missense mutation of *Foxp2* yields an arginine-to-histidine substitution in the DNA-binding domain of the encoded protein, matching a human FOXP2-R553H mutation which causes speech and language problems in the large well-studied KE family [18]. The S321X nonsense mutation of *Foxp2* results in an early stop codon at position 321 of the protein, close to a human FOXP2-R328X mutation impairing speech and language in a second smaller family [19]; both the mouse S321X and the human R328X mutations are likely to represent null alleles [12], [19]. We tested mice on the C3H background as a) wildtypes (WT), b) mice heterozygous for the R552H missense mutation (R552H), and c) mice heterozygous for the S321X nonsense mutation (S321X). Each group of animals consisted of 11 females at the age of 8 weeks at the beginning of the experiments. As in the affected humans, we investigated the mouse mutants in the heterozygous state. In the homozygous state these mouse mutations cause general developmental delays, severe motor impairments and postnatal lethality prior to weaning – no humans with homozygous mutations have ever been reported [12].

**Figure 1:**
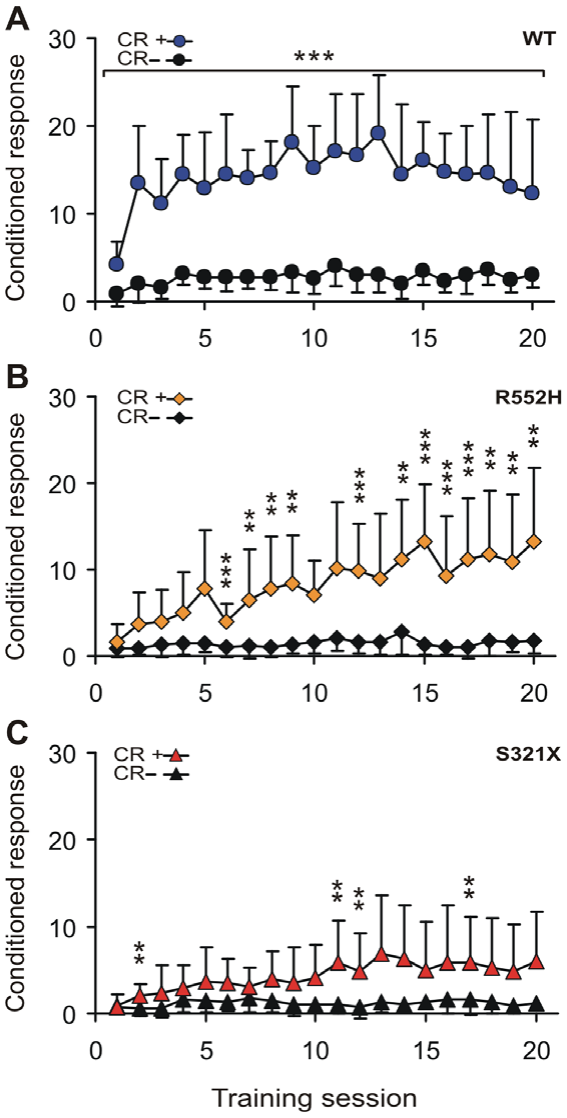
Learning curves of wildtype (a), heterozygous R552H mutant (b), and heterozygous S321X mutant (c) mice. For all 20 training days (one training session per day) the mean numbers of jumps across the hurdle averaged from the performances of the 11 animals per experimental group are shown. The animals could show hits (CR+) in the presence of 12 kHz tones or false alarms (CR−) in the presence of 7 kHz tones. Since each training session consisted of 60 trials with 30 randomized presentations of both CS+ and CS− a maximum of 30 hits and 30 false alarms could be reached if the animals responded to each tone with a jump, irrespective of the tone frequency. The larger the distance is between the CR+ and CR− curves the better is the learning performance. Standard deviations of the means are shown only for one side to improve readability of the data. Statistically significant differences between the CR+ and CR− rates calculated for each training session are indicated as ** p<0.01; *** p<0.001.

The learning curves of the three genotypes in Fig. 1a–c show how the performance of the animals changed over the 20 training sessions. A learning effect is indicated by the increase of the number of hits (CR+, i.e. hurdle crossings as conditioned responses to the 12 kHz tones) relative to the number of false alarms (CR−, i.e. hurdle crossings to the 7 kHz tones which actually require that the animal remains in the compartment where it is). Prominent learning differences between the genotypes become evident. WTs performed significant tone discrimination, indicated by asterisks in the figure, from the first training day onwards (Fig. 1a), R552Hs from day 6 onwards (with non-significant values at days 10–13; Fig. 1b), and S321Xs only sporadically (Fig. 1c).

**Figure 2:**
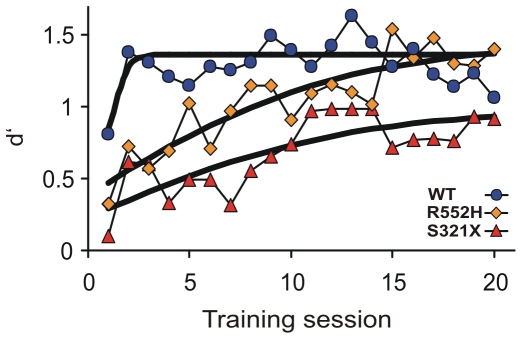
Logistic growth functions modeling the increase of the discrimination index d′ as function of the training day. d′ expresses the achieved average performance level of tone discrimination of the animals in each experimental group (see Methods). Discrimination performance of the WTs increases rapidly and stays at a maximum level already from day 2 onwards. Discrimination performance of the R552H heterozygotes increases slowly but finally reaches the level of the WTs. Discrimination performance of the S321X heterozygotes increases very slowly and does not reach the levels of WTs and R552H heterozygotes. The correlation coefficients of the growth functions are statistically significant (p<0.01 in each case).

Learning curves can be expressed by logistic growth functions of the discrimination index, d′ [29]. Figure 2 shows such functions which approximate the data from the three genotypes with statistically significant correlation coefficients (p<0.01). The functions show (Fig. 2) that WT animals learned rapidly, the R552H genotype learned slowly but reached the same performance level as WTs after about 15 training days, while S321X animals learned very slowly and remained in their discrimination performance significantly below the level of WTs and R552Hs.

**Figure 3:**
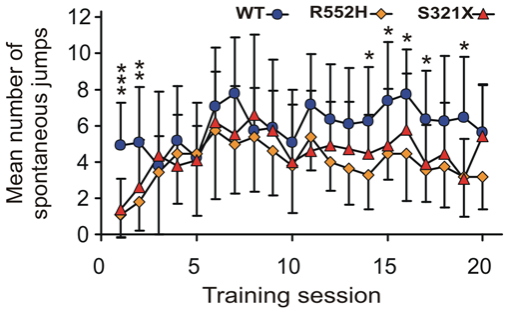
Spontaneous motor behavior. Mean numbers of spontaneous jumps across the hurdle of the shuttle-box during the three minutes before the beginning of the daily training session. At the first training day, WTs show significantly more spontaneous jumping compared to both types of heterozygous mutants (*** p<0.001 in each case; F-value of the ANOVA = 14.92). At the second training day, the WTs show significantly more jumps than the heterozygous R552H mutants (p<0.02 **; F-value of the ANOVA = 5.12). For training days 3–13, 18 and 20 the ANOVA-tests did not lead to significant differences, F<3.42). WTs showed more spontaneous jumps compared to both mutants (p<0.05*; F>5.30) on days 17 and 19, and compared to R552H mutants (p<0.05*; F>4.40) on days 14–16. Standard deviations of the means are shown only for one side to improve readability of the data.

**Figure 4:**
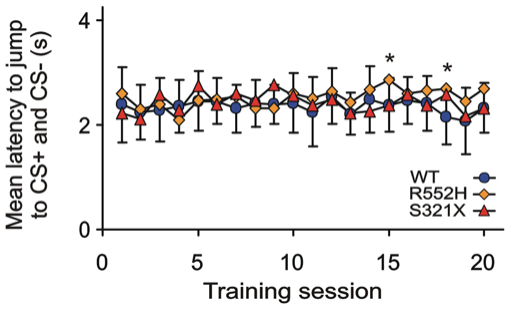
Latencies of jumps across the hurdle. Mean latencies of jumps across the hurdle of the shuttle-box after tone onset are plotted as a function of the training sessions. There are no systematic significant differences of latencies between the experimental groups of animals. At training session 15, R552H mutants are significantly different from WTs and S321X mutants (p<0.05; F = 4.42) and at training session 18, WTs differ from R552H mutants (p<0.05; F = 5.41). At any other training session, significant differences did not occur (F-values<2.50). Standard deviations are shown only for the WTs for better visibility. They are of the same order of magnitude for both groups of heterozygous mutants.

The initial rapid performance improvement of WTs during the first two days of conditioning (Figs. 1a, 2) reflects excellent procedural learning [28] which is absent in both types of mutants (Figs. 1b–c, 2). An important part of the learning procedure is the jump across the hurdle in the shuttle-box. We measured the rate of spontaneous hurdle crossings during the three minutes before the beginning of the daily training session. Interestingly, both mutants showed a significantly lower rate of spontaneous hurdle crossings compared to WTs at the first training day, R552H heterozygotes also at the second training day (Fig. 3). Because of the large standard deviation of the mean of the S321X heterozygotes, they did not differ, according to our criterion, from the WTs at the second day. The deficit in spontaneous hurdle crossings of both heterozygous mutants was not due to hesitation or anxiety to jump because if mutants jumped in response to the presented tones at all, they jumped with the same latency as the WTs on all training days (Fig. 4). There may be two reasons for the lower rates of spontaneous hurdle crossings of heterozygous mutants during the first two days. (1) The Foxp2 mutation could have reduced the exploratory behavior in an unknown (first day) or not yet well known (second day) environment, and/or (2) the mutation may have prevented the mice during their exploratory behavior from jumping across the hurdle, because they observed an obstacle not easily to be crossed. Since heterozygous mutants do not differ from WTs in spontaneous locomotor activity and exploratory behavior in an elevated plus-maze and a T-maze [12], we favor the second explanation, i.e. on the first two training days, the hurdle was an obstacle difficult to be traversed and only the pressure of the training paradigm led the heterozygous mutants to acquire the motor skill of jumping, which then served as basis for further auditory-motor learning. From training day 3 onwards, spontaneous hurdle crossings occurred at similar rates in WTs and both heterozygous mutants (Fig. 3), so that deficits in motor skills cannot explain the differences in discrimination performance between the different groups of animals evident in Figs. 1a–c, 2 after the second training session. Furthermore, small differences in auditory sensitivity between the R552H and S321X mutants and the WTs detected in measurements of auditory brainstem potentials [30] are irrelevant for the perception of the frequency differences presented here, because the frequency discrimination task (12 vs. 7 kHz) is far above the frequency discrimination limens [31]. Following the interpretation of the shape of learning curves of mice in the shuttle-box [28], we propose that the most convincing causes of the discrimination deficits of the mutants compared to the wildtype animals after the second training session are problems with auditory-motor association learning, being especially severe in the S321X mutants (Figs. 1c, 2).

In a previous study of heterozygous Foxp2 knockout mice using the Morris water maze [32], the knockout animals displayed equal performance levels to WTs over the whole test period (8 days), indicating that they had intact abilities to associate visuospatial cues with the orientation of their own swimming movements. Such findings suggest that heterozygous disruption of *Foxp2* does not have general effects on learning of sensory-motor associations and handling these associations in working memory. Also of relevance are results from a recent study of the KE family [33] who have a speech and language disorder caused by the R553H mutation of human FOXP2, corresponding to the R552H mutation of mouse Foxp2. Affected KE family members were reported to have significant deficits in phonological working memory as compared to unaffected members, but did not show differences in their general working memory or on tests of visuospatial association [33]. The authors propose that disruption of FOXP2 in humans may specifically affect the ‘motor-related representations required for internal rehearsal of speech-based material in phonological working memory’ [33]. Our own results predict that humans affected by FOXP2 mutations may have underlying deficits in auditory-motor association learning, and that these could be contributing to their difficulties with developing fluent speech.

The R552H mutant allele yields a full-length Foxp2 protein carrying a substitution in its DNA-binding domain. This mutant protein has significantly disturbed transcription factor function but it appears stable and can still interact with other Foxp proteins present in the cell, including the WT Foxp2 protein in heterozygotes [34]–[36]. In contrast any mutant Foxp2 protein encoded by the S321X mutant allele would be dramatically truncated, and primarily located in the cell cytoplasm, rather than the nucleus [34], [35]. Moreover, the S321X allele may be subject to nonsense-mediated RNA decay and protein instability, making it effectively null [12]. Therefore, the present data suggest important differences in the consequences of two different mutations of the *Foxp2* gene on learning behavior. In our study, motor-skill learning was similarly disturbed by both types of mutation, while auditory-motor association learning was more severely affected by the null allele than by presence of a dysfunctional protein.

In summary, our study of etiological *Foxp2* mutations in mice has revealed novel cognitive deficits that go beyond motor functions and extend to auditory-motor association learning. The effects of this gene on learning processes are consistent with previous demonstrations that it regulates the expression of target genes involved in neurite outgrowth and synaptic plasticity [5], [37]–[39]. This work opens up a new perspective for understanding how disruptions of *FOXP2* lead to disordered speech and language development. In humans such mutations may affect not only the sequencing of articulatory gestures necessary for fluent speech, but also the ability to associate auditory percepts with the corresponding motor programs for vocal imitation [33] as found in a songbird model [11]. The phenotypic differences we observed between the mutation types in mouse models suggest the existence of, so far undescribed, differences in learning performance in humans with distinct *FOXP2* mutations.

## Materials and Methods

### Animals

Both mutant mouse lines were originally generated via a gene-driven N-ethyl-N-nitrosourea (ENU) mutagenesis strategy [12]. As previously described, the founders were crossed onto the C3H/HeNHsd background for up to nine generations, exploiting marker-assisted backcrossing to accelerate homogenization of genomic background and elimination of non-relevant ENU mutations [12]. The behavioral experiments were carried out in accordance with the European Communities Council Directive (86/609/EEC) and approved by the Regierungspräsidium Tübingen, Germany (numbers 846 and 1050). Data were obtained from female mice, 11 wildtype C3H/HeN (WT), 11 heterozygotes R552H (R552H), and 11 heterozygotes S321X (S321X). We tested female mice because we know from tests on NMRI mice [28] and other strains (unpublished data) that females are more cooperative in this sort of learning paradigm and, thus, reach higher average performance scores and less variable data compared to males. Therefore females are better indicators of possible changes in learning performance in the shuttle-box and, thus, are better suited for our present tests than males.

The WT group contained 5 littermates of the heterozygous mutants and 6 further WTs of the same strain. The learning curves of both WT subgroups did not differ over the whole 20 days of testing and were pooled. At the beginning of the experiments all animals were 8 weeks old. Animals were housed in same-sex groups in standard laboratory cages with free access to food (rodent pellets) and water at an average temperature of 22°C and a 12 h light-dark cycle (light on at 7 AM).

### Apparatus, Training Procedure and Behavioral Measures

Animals were trained in one daily session in a two-compartment shuttle-box using a go/no-go avoidance discrimination learning procedure. The shuttle-box (Coulbourn Instruments, Whitehall, USA) had a hurdle of 2.5 cm height in its center separating the two compartments. Mice had to cross the hurdle in response to tones of a given frequency or stay in the compartment where they were in response to tones of another frequency. The sound stimuli were digitally synthesized pure tones (44.1 kHz sampling rate, 16-bit dynamic range) of 400 ms duration (5 ms rise and fall times included) and 2 Hz repetition rate. The tones were delivered through two loudspeakers, one at each top of the two compartments of the shuttle-box. The sound pressure levels were calibrated to 70+/−5 dB at the floor level of the shuttle-box (microphone, microphone power supply, amplifier: Brüel & Kjaer 4135, 2633, 2636, respectively). 12 kHz tones were the conditioned stimulus that should initiate a go-response (CS+, jump over the hurdle) while 7 kHz tones should initiate a no-go-response (CS−, stay in the compartment where you are). The acoustic properties of the tones allowed perception at about 50 dB or 55 dB sensation level for the 7 and 12 kHz tones, respectively, as derived from behavioral tests of absolute auditory thresholds [40]. Hence, both tones were perceptible far above the absolute hearing threshold and were clearly audible stimuli. Further, auditory brainstem response audiometry [30] showed that animals of all three experimental groups used here had very similar hearing sensitivity in the frequency range of the present tones. Finally, the 7 and 12 kHz pure tones used in our paradigm do not occur in the vocal repertoire of the mouse [41] and, thus, have no inherently special meaning in mouse communication. Therefore, there are no differences in audibility or salience of the two tones used here for conditioning of tone discrimination that could be responsible for the performance differences between the groups.

As in a previous study [28], each training session consisted of 60 trials with 30 randomized presentations of both CS+ and CS−. Inter-stimulus intervals had durations of 15 s. Electrical foot shocks of 70–120 µA applied through the floor grid served as unconditioned stimuli (UCS). To achieve a mild escape response in the animals, the shock level was adjusted individually. The animals learned to avoid the foot shock by making a decision about crossing of the hurdle within 4 s after the onset of one of the sounds to be discriminated. The animals could show four types of responses to the CS+ and CS− presentations: a) Hurdle crossing within 4 s after onset of the CS+ was considered a ‘hit’ (conditioned response CR+). The CS+ presentation was stopped as soon as the hurdle was crossed and no UCS was delivered. b) A ‘miss’ was noted when the animal did not cross the hurdle within 4 s after the onset of the CS+. In that case, the CS+ was continued together with an UCS presentation for maximally another 4 s in order to motivate the animal to cross the hurdle. c) A ‘false alarm’ (CR−) was noted when the animal crossed the hurdle during the 4 s CS− presentation. In that case the animal received an UCS in the compartment to which it had crossed (0.5 s error-shock). d) A ‘correct rejection’ was noted when the animal remained in the compartment during the 4 s presentation of the CS−. The only difference to the previous study [28] was the training over 20 days (not 15 days as before) in order to measure possible late improvements of performance in the slowly learning mutants.

In addition to the decisions of the mice in response to the presentation of the CS+ and CS− stimuli, two further measures of the behavior of the animals were taken. After an animal was put into the shuttle-box, it had three minutes without stimuli in order to get accustomed to the situation. During this time, the animal could move freely around and cross the hurdle between the two compartments of the box. We noted all spontaneous hurdle crossings during the three minutes. Further, the latency from the start of a sound (CS+ or CS−) to the jump over the hurdle (hind legs lose contact with the compartment from which the jump was initiated) was measured for all jumps of all animals.

### Data Analyses

All data about stimuli, responses and reaction times were automatically recorded to disk and stored for off-line computer analysis. All statistical tests were done with STATISTICA (version 9.1 by Statsoft) with α = 0.01. Separately for every training session and experimental group, means with standard deviations of CR+ and CR− rates were calculated (Fig. 1a–c) and tested for statistical differences (CR+ vs. CR− rates; Mann-Whitney U-test, two-tailed). According to signal detection theory [29], the discrimination index d′ was calculated for each group (d′ = z (CR+rate)−z (CR−rate)). The development of the d′ function over the training sessions (Fig. 2) represents logistic functions with the equation d′ = A+C 1/(1+e^–B(x – M)^) in which A defines the lower asymptote = minimum, B the growth rate, C the upper asymptote together with parameter A (the maximum = A+C), M the x-value of the inflection point (maximum slope). The logistic functions approximate the data points with statistical significance (one-tailed) of the regression coefficients (r). WT: r = 0.5452 (p<0.01); R552H: r = 0.8958 (p<0.001); S321X: r = 0.7830 (p<0.001). The mean numbers of spontaneous jumps (Fig. 3) and the mean latencies to jump (Fig. 4) were tested for significant differences between the groups with a one-way ANOVA (two-tailed) and post-hoc group comparisons (Tukey-test).
